# Vertical transmission of gut bacteria in commercial chickens is limited

**DOI:** 10.1186/s42523-023-00272-6

**Published:** 2023-10-10

**Authors:** Naama Shterzer, Nir Rothschild, Yara Sbehat, Jonathan Dayan, Dor Eytan, Zehava Uni, Erez Mills

**Affiliations:** Department of Animal Sciences, Robert H. Smith Faculty of Agriculture, Food, and Environment, The Hebrew University of Jerusalem, 7610001 Rehovot, Israel

**Keywords:** Chicken, Gut microbiota, Vertical transmission, Chick colonization, Interventions

## Abstract

The existence of vertical transmission in chickens under commercial settings, where chicks are raised separately from adults, is unclear. To answer this question, the fecal microbiota of chicks hatched and grown separately was compared with their mothers’ microbiota. Most amplicon sequence variants (ASVs) identified in hens were not detected at all in chicks up to two weeks of age by 16S rDNA sequencing, and those that were detected had a low incidence among the chicks. Nevertheless, a few ASVs that were common with the hens were highly prevalent among the chicks, implying that they were efficiently transmitted to chicks. These ASVs were culturable from the reproductive tract of hens and eggshells. Furthermore, interventions attempting to disrupt transmission resulted in a reduction in the prevalence of specific phylogenetic groups in chicks. To conclude, vertical transmission in commercial poultry grown separately from adults likely exists but is not efficient, possibly resulting in impairment of microbiota function. This implies that artificial exposure to adult bacterial strains might improve microbiota functioning.

**Supplementary Information:**

The online version contains supplementary material available at 10.1186/s42523-023-00272-6.

## Introduction

The gut microbiota provides important functions for the host, including protection from pathogens [1], breakdown of plant-derived nutritional fibers, and signals that regulate the maturation and development of host systems, including the immune, neurological and intestinal systems [2]. Thus, efficient vertical transfer of gut bacteria from parents to offspring is important for the establishment of optimal microbiota functioning early on. Efficient vertical transfer is also important for gut microbes, especially when considering the advantages of early colonization of a niche [3].

In nature, hens brood on their eggs, and after hatching, the chicks will stay with the hen for a while. Cohabitation, or direct contact between parents and offspring, is an important mechanism for vertical transmission in animals, including poultry [4, 5]. Gut bacteria can also possibly utilize the egg for vertical transfer. Pathogens such as *Salmonella* are known to use the egg for vertical transmission [6, 7]. A vertical transmission mechanism utilizing the egg might be important in commercial settings where fertilized eggs are separated from the hens immediately after being laid, as well as for other vertebrates in which the parents leave the fertilized eggs after laying them and never interact with their offspring. A third mechanism of vertical transmission might be the indirect long-term survival of microbes in the environment as they wait to be picked up by a randomly passing newly hatched chick. It should be noted that in many commercial operations, eggs and other surfaces are disinfected by a variety of chemical treatments aimed at inhibiting pathogen transmission [8]. Thus, it is unclear whether vertical transfer of gut bacteria in commercial chickens still occurs or has been partially or fully severed.

A number of reports imply the existence of vertical transmission through the egg in poultry. First, as already mentioned, pathogens use poultry eggs for transmission [6, 7]. Furthermore, a reproductive tract microbiota was shown to exist [9], and a large overlap in the intestinal and reproductive tract microbiomes of chickens was found. A correlation between the levels of relative abundance of bacteria in the gut and their chance of being present in the reproductive tract has been shown, implying that gut contents are sampled into the reproductive tract and that gut bacteria are in the right place to integrate into the forming egg [10]. Moreover, an embryo gut microbiota has been implied by 16S rRNA gene sequencing [9], and some similarity at the genera level between hens and their offspring has been shown [11]. Last, it has been shown that both the eggshell and the environment contribute to the intestinal microbiota of growing chicks [12].

However, it has also been shown that exposure of newly hatched chicks to a hen, even for just 24 h, greatly affects the gut microbiota of chicks [4]. Additionally, exposure of newly hatched chicks to adult gut contents has been shown to be protective against *Salmonella* infection [13, 14], further implying that microbiota functions were lacking in newly hatched chicks, possibly because of lack of exposure. Thus, it is unclear if vertical transmission of gut bacteria occurs in commercial chickens, and if the chicken egg supplies not only nutrients but also commensal gut bacteria for the developing chick. Furthermore, vertical transmission might be different for different bacterial phylogenetic groups depending on their specific mechanism of vertical transmission.

Here we compared the fecal microbiota of hens and their specific progeny using 16S rRNA gene sequencing at the Amplicon Sequence Variant (ASV) level during three cycles of chick growth to understand whether vertical transmission occurs in commercial chicks growing apart from adults. We rated different ASVs by their incidence in the chick population, indicating their ability to transmit to chicks. We also cultured ASVs from the hen reproductive tract and eggshells to unravel the mechanism of vertical transmission. Last, we disrupted possible vertical transmission by either disinfecting the eggshell or by giving an antibiotic cocktail to the hens during the week of egg collection.

## Materials and methods

### Experimental outline and ethics

All animal trials were conducted in accordance with the guidelines of the National Council for Animal Experimentation and were subjected to approval by the Hebrew University of Jerusalem’s Ethics committee, approval No. AG-18-15514-3 and AG-19-15897-3.

**Fig. 1:**
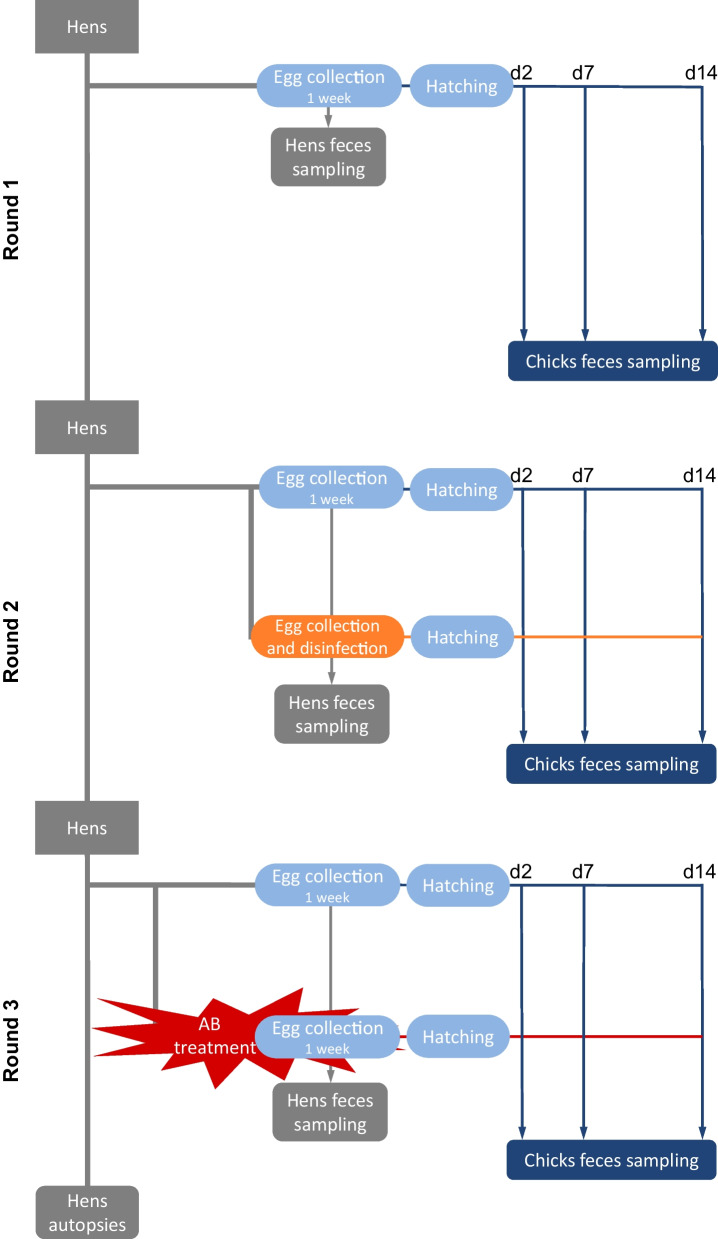
Experimental outline. In the first round of the experiment, fertilized eggs were collected for a week from ten broiler breeder hens and incubated until hatching. Individual fecal samples were collected from the hens during the egg-collection week. Chicks were raised for 14 days, and individual chick fecal samples were collected on days 2, 7 and 14. This was repeated twice more with the same hens, with a different intervention performed on half of the hens or their eggs in each round. In the second round of the experiment, eggs laid by half of the hens were disinfected before incubation, while eggs from the other hens remained untreated. In the third round, half of the hens were treated with an antibiotic cocktail for two weeks, including the egg-collection week. Internal organs were sampled from the hens at the end of the experiment

In the first round of the experiment, eggs were collected for a week from ten broiler breeder hens of the Ross breed that were housed in the experimental farm at the Hebrew University of Jerusalem’s Faculty of Agriculture and incubated until hatching (Fig. 1). Individual fecal samples were collected from the breeder hens during the week of egg collection. Chicks were raised for 14 days, and individual fecal samples were collected on days 2, 7 and 14. This experiment was repeated twice more with the same hens, with a different intervention performed on half of the hens in each round. In the second round of the experiment, eggs laid by half of the hens were disinfected prior to incubation, while eggs from the other hens remained untreated. In the third round, half of the hens were treated with an antibiotic cocktail for two weeks, including the egg-collection week. Of note, the hens treated in the third round with the antibiotic cocktail are the same hens that had their fertilized eggs disinfected in the second round. A full description of both interventions is found below. All hens were euthanized at the end of the experiment, and samples of internal organs (jejunum, cecum and magnum) were collected.

### Hens’ growth conditions

Female and male Ross broiler breeders (n = 10 each), 37 weeks old, of commercial origin were placed in the experimental chicken house at the Hebrew University of Jerusalem’s Faculty of Agriculture. The animals were acclimatized for six weeks before the experiment. Broiler breeders were housed in individual cages and kept according to the Ross Parent Stock Management Handbook. The feed used in the experiment did not contain antibiotics. Fresh semen was collected and pooled from all the males and used to artificially inseminate all the females every 5 days. Egg collections of rounds 1, 2 and 3 were performed when the hens were 43, 49 and 53 weeks old, respectively.

### Egg incubation and chick growth conditions

Eggs were collected with clean gloves, placed in clean and disinfected plastic trays, and kept at 18 °C for up to 7 days before incubation. Incubation was performed in clean and disinfected Maru 190 Delux incubators (Rcom, Korea) for 21 days at 37.8 °C and 58% relative humidity, with automatic rotation every hour. Each clutch of eggs was incubated in a separate incubator. On incubation day 18 rotation was stopped, and the eggs were placed in hatching trays separated by clean and disinfected individual metal cages.

After a 24 h hatching period, chicks were moved to clean and disinfected plastic containers (2,368 cm^2^, up to 7 chicks per bin) with sawdust bedding, equipped with two feeders and two water bottles. Chicks had ad libitum access to feed and water and were raised according to the Ross Broiler Management Handbook. The only exception was that chicks received the same feed as the adults. This was to remove the possibility that differences in the feed would affect the ability of hen-derived bacteria to grow in the chick gut.

Chicks from interventions and control chicks were kept in separate containers in the same room. The first round included 41 chicks. The second round included 22 chicks from disinfected eggs and 25 control chicks. The third round included 20 chicks from antibiotic-treated hens and 23 control chicks.

### Eggshell disinfection and sampling

Disinfection was aimed at disrupting the transmission of bacteria that might utilize the eggshell to transmit to chicks. During the second round, within 24 h of laying, eggs were thoroughly sprayed with 0.3% Virocid (CID LINES, Belgium), which was preheated to 45 °C, and were then left to dry for 10 min. After drying, the eggs were sprayed again in the same manner and left to dry for another 10 min. A 2 × 2 cm area of each egg was sampled using a sterile flocked swab (Deltalab, Spain) slightly moistened with Difco D/E neutralizing buffer (BD, France). The swab was then directly suspended in 600 μl of the buffer. The suspension (200 μl) was plated on both aerobic and anaerobic YCFA plates [15]. Plates were incubated for two days at 37 °C, and CFUs were counted.

### Antibiotic cocktail treatment

Treatment with an antibiotic cocktail was aimed at disrupting the gut and reproductive tract microbiota of the hens and thus disrupting direct transmission from hens to chicks, whether bacteria were using the eggshell or the egg content to transmit to chicks. In round three, five hens (mean body weight 4.02 ± 0.3 kg) were orally gavaged twice daily for two weeks with an antibiotic cocktail containing a total of 150 mg/day Ampicillin, 100 mg/day Colistin, 400 mg/day Neomycin and 400 mg/day Metronidazole. These drugs represent different classes of antibiotics, directed against different targets in the bacterial cell, thus covering a wide spectrum of bacteria [16–19]. Furthermore, these antibiotics were found to leave no residues in eggs when administered orally to laying hens [20].

### Sample collection

Chick fecal samples were collected within minutes by individually placing each chick on a clean piece of paper; hen fecal samples were collected within an hour by placing a clean plastic sheet beneath each cage. Fresh fecal samples were transferred into 5 ml of sterile PBS, snap-frozen in liquid nitrogen and kept at − 20 °C until DNA extraction.

At the end of the experiment, the hens were euthanized, and GI tract and reproductive tract samples were removed. Contents were pressed out of the GI tract samples, and mucosa was scraped with a sterile glass slide from the magnum section of the reproductive tract. Samples were mixed with 5 ml sterile PBS, snap-frozen in liquid nitrogen and kept at − 20 °C until DNA extraction.

Eggshell and reproductive tract samples used to check for viability of bacteria were obtained from 40-week-old Cobb broiler breeders (n = 10), as described previously [10]. Reproductive tract samples were mixed with 5 ml sterile anaerobic PBS + 10% glycerol, snap-frozen in liquid nitrogen and kept at − 80 °C until use. To plate the reproductive tract samples, the entire sample was centrifuged (12,000 g, 2 min) in anaerobic conditions. The pellet was washed with PBS, centrifuged again and finally resuspended in 150 µl PBS, which were all plated on an anaerobic YCFA plate [15]. Nine eggs were collected from the broiler breeders during 48 h prior to internal sample collection. Egg swabs were plated on aerobic and anaerobic YCFA plates. All plates were incubated at 37 °C for three days, and all of the colonies were collected in batch from each plate into sterile PBS for subsequent DNA extraction and 16S rRNA gene sequencing.

### DNA extraction and 16S rRNA gene sequencing

DNA was extracted as described previously [10]. Briefly, samples were disrupted with 0.1 mm glass beads in the presence of Tris-saturated phenol, followed by phenol–chloroform extraction, as described by Stevenson & Weimer [21]. Isopropanol was used to precipitate the DNA.

16S rRNA gene library preparation and sequencing were performed according to the Earth Microbiome Project protocol [22] using V4 primers 515F (GTGYCAGCMGCCGCGGTAA) and 806R (GGACTACNVGGGTWTCTAAT). Paired-end sequencing (150 bp) was performed on an Illumina MiSeq platform using a V2 reagent kit by the sequencing unit of the Faculty of Medicine at the Hebrew University of Jerusalem. Sequences were processed and taxonomy assigned using QIIME2 [23]. ASVs were determined with Dada2 plugin version 2018.8.0 [24] using the denoise-paired method. ASVs with under 5 reads were discarded. All samples were normalized to 5,000 reads per sample, except eggshell plating samples, which were normalized to 3,800 reads per sample. Taxonomy was assigned using a naive Bayes classifier [25] trained on the Silva 138 database [26]. The feature table and taxonomy assignments can be found in Additional file 2.

### Transmission index

Transmission index was calculated for each ASV in each round as the percentage of chick fecal samples containing the ASV out of the total number of chick samples in that round.

### Statistical analysis

All statistical analyses were performed using GraphPad Prism 8.0.0 (GraphPad Software, San Diego California USA, www.graphpad.com), with the exception of ANOSIM tests, which were performed using Past 4.05 [27]. The significance of the ratios between treated and untreated chicks was inferred by shuffling the treated/untreated labels of the samples and calculating the ratios 1,000 times and then comparing the real value to the distribution of permuted data. Values were considered significant if they were more than two standard deviations from the mean. Only phylogenetic groups represented by at least 60 occurrences in chick fecal samples were analyzed.

## Results

### The fecal microbiota of 14-day-old chicks is different from that of adult hens

**Fig. 2:**
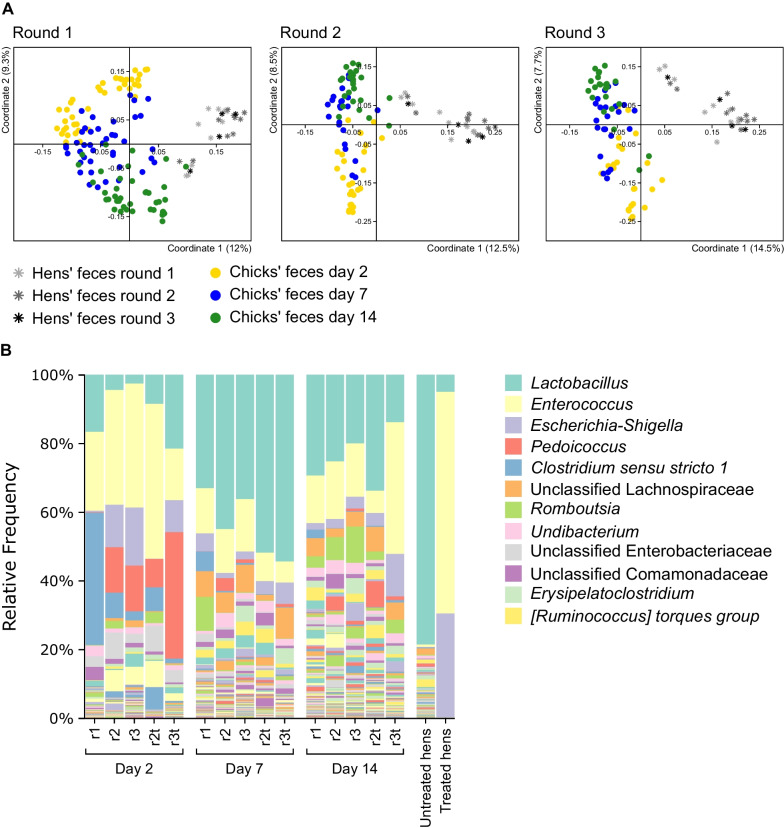
Microbiota composition of chicks and hens. **A** PCoA analysis of untreated chicks and hens in different rounds based on Jaccard index. Each of the three insets displays all untreated hen fecal samples but only the round specific untreated chick fecal samples. **B** Taxonomic composition of all fecal samples at the genus level. r1 = round 1; r2 = round 2; r3 = round 3; r2t = round 2 treated chicks; r3t = round 3 treated chicks

As a first step, we confirmed that the fecal microbiota of untreated chicks up to the age of two weeks was different from that of adult hens. It should be noted that for broilers, 14 days constitute approximately one-third or more of their lifespan and that for both broilers and layers, the first week of life is considered important for overall growth as well as sensitivity to pathogens [28]. An analysis by Jaccard index showed that the fecal microbiota of untreated chicks changed from day 2 to 14 but that on day 14, it was still different than that of adult hens (Fig. 2A, Additional file 3: Table S1). Furthermore, an analysis of relative abundances showed that the fecal microbiota of adult hens was dominated by the genus *Lactobacillus*, which accounted for 78.6% of the fecal community (Fig. 2B). While this genus was also an important genus in the microbiota of untreated chicks, it comprised, on average, just 7.9% on day 2, 38.1% on day 7 and 24.9% on day 14. Of note, because the chicks of the three rounds came from the same hens and were housed in the same environment, we hypothesized that the chicks’ fecal microbiota would be similar. However, a comparison of the untreated chicks in the three rounds showed that the chicks’ fecal microbiota at each of the three time points was different between rounds (Additional file 3: Table S2), whereas the hens’ fecal samples were not significantly different between rounds (Additional file 3: Table S3). Thus, even though they originated from the same hens, the untreated chicks of the three rounds had a different fecal microbiota. To conclude, after 14 days the chick’s fecal microbiota was still different from that of an adult, and a round-specific effect on composition was identified.

### After 14 days of life the chick fecal microbiota lacks most of the hen fecal ASVs

**Fig. 3:**
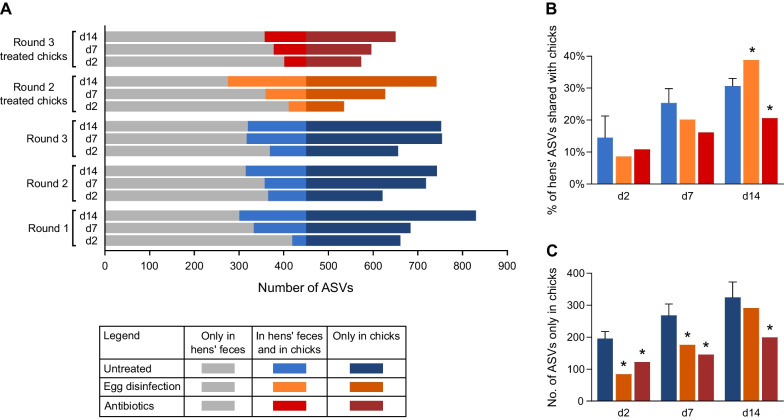
Analysis of hens’ and chicks’ shared and non-shared ASVs. **A** All untreated hen fecal samples were pooled and compared to the pooled chick samples of each specific day and round. Number of ASVs present only in hens' feces (gray bars), in hens’ feces and in chicks (light color bars) and only in chicks (dark color bars) are presented. **B**, **C** Percent of hens’ ASVs that also appear in chicks (**B**) and number of ASVs that appear only in chicks (**C**). Data are presented as the mean ± SD, for the untreated group consisting of all three rounds; egg disinfection and antibiotics groups were present only in one round, thus their data presents only the mean. Two-tailed one-sample t-test against data of all rounds (n = 3) of untreated chicks; *p < 0.05

One possible cause for the difference in the microbiota of chicks and hens could be that chicks lack the bacterial strains found in hens. To determine if this is the case, we classified each ASV in our data as unique to hens, unique to chicks or shared by both. We compared only ASVs found in the hen fecal matter to ASVs found in chick samples, as only fecal samples were collected for the chicks. It was found that most of the hens’ fecal ASVs were not identified in chick samples using 16S rRNA gene sequencing (Fig. 3A). This implied that most hen ASVs did not reach chicks growing in commercial conditions, during which there is no interaction between hens and chicks.

The percentage of hen ASVs found in the fecal samples of the whole chick flock increased from 14.6% on day 2 to 30.7% on day 14 (Fig. 3B). Notably, the difference between day 7 and day 14 was smaller than that between day 2 and day 7. One hypothesis is that over time, the flock is exposed to more ASVs shared with the hens, which then colonize the chicks. Another possibility is that most of the added shared ASVs already colonized some of the chicks at hatch but did not pass the 16S rRNA gene sequencing sensitivity threshold on day 2.

### Most chick ASVs that are shared with the hen do not efficiently transmit to chicks

**Fig. 4:**
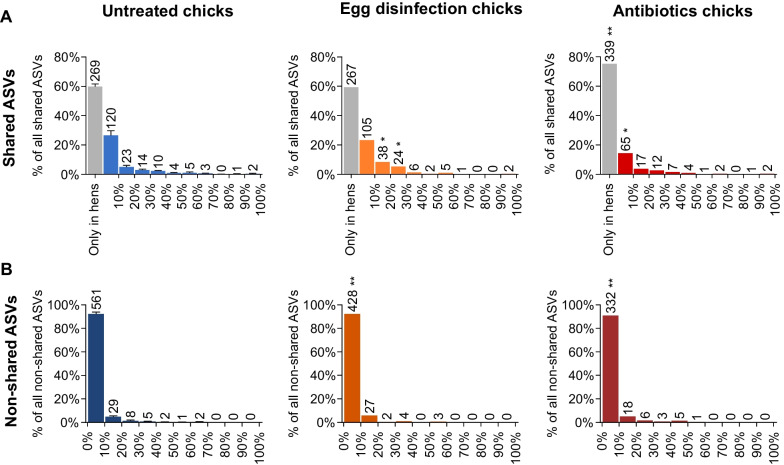
Analysis of transmission scores for all ASVs. All hen ASVs (**A**) and chick ASVs not appearing in hens (**B**), binned by their transmission score. Gray bars in (**A**) represent hen ASVs that did not appear at all in chicks (transmission score = 0%). Data are presented as the mean ± SD, for the untreated group consisting of all three rounds; egg disinfection and antibiotics groups were present only in one round, thus their data presents only the mean. The number of ASVs in each bin in indicated above the bar. Each bin of the treated chicks was compared with its counterpart of the untreated chicks (n = 3) by two-tailed one-sample t-test; *p < 0.05, **p ≤ 0.01

While the analysis of shared ASVs, described in the previous section, already showed vertical transmission to be inefficient, it is in fact a permissive analysis because an ASV was considered shared even if it was found only once among all chick fecal samples. To better quantify the ability of specific ASVs to transmit to chicks, a “transmission score” was calculated. The transmission score is the percentage of chick samples in which a certain ASV was identified. For this analysis, fecal samples from days 2, 7 and 14 were counted together because we hypothesized that efficiently vertically transmitted ASVs were likely to be identified in day 2 fecal samples, while less efficiently transmitted ASVs would appear later on. An analysis of transmission scores showed that most hen ASVs that were also identified in the chicks were found in less than 30% of the samples and therefore were poorly transmitted to chicks (Fig. 4A). Only 4.89 ± 1.34% of the hen fecal ASVs were found in 30–70% of the chick fecal samples, implying that they had an intermediate ability to transmit to chicks. In all three rounds, only three ASVs, which account for 0.67% of all hen fecal ASVs, had a transmission score higher than 70%. To conclude, using 16S amplicon sequencing and our specific primer set, most ASVs found in the hens were not identified at all in the chicks, and of those that were identified, most were poorly transmitted to chicks.

### Three ASVs were efficiently transmitted to chicks

**Fig. 5:**
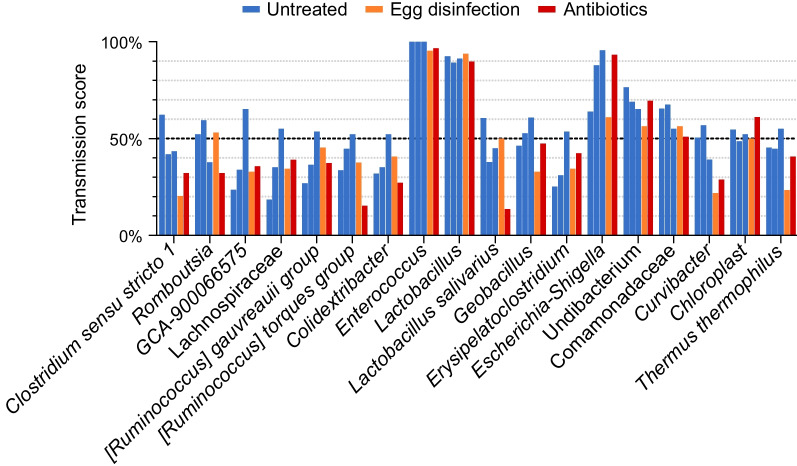
Intermediate and efficiently transmitted ASVs in chicks. Transmission score of the top scoring ASVs among ASVs shared with hens. All ASVs with a transmission score of ≥50% in at least one round are presented. The highest level of informative taxonomic annotation obtained for each ASV is denoted. Transmission score in three rounds of untreated chicks (blue), egg disinfection (orange) and antibiotics (red) are presented

While most hen ASVs were poorly transmitted to chicks, there were a few exceptions. Three ASVs were consistently transmitted to chicks efficiently in all three rounds of the experiment (Fig. 5). These were annotated taxonomically as unclassified *Escherichia-Shigella*, *Enterococcus*, and *Lactobacillus*. It should be noted that most top scored transmitted ASVs, including these three as well as those with intermediate transmission scores, were consistent in the three rounds (Fig. 5). This implied that transmission of these ASVs to chicks was not stochastic but rather the result of their biology and their interactions with the host and the environment. Interestingly, a comparison of transmission scores of all ASVs with their relative abundance in the chick fecal samples showed a good correlation (Additional file 1: Fig. S1). Thus, the ability to spread in the flock was connected to the ability to compete and reach high numbers in the microbial community. In fact, 75.3% of the chick samples were dominated by a single ASV, which accounted for 30% or more of the relative abundance, and in 70.3% of them this was one of the three efficiently transmitted ASVs (Additional file 1: Fig. S2). To conclude, we found ASVs common with the hens that were consistently transmitted to chicks. These ASVs are either transmitted through the egg or are good at surviving in the environment, colonizing the newly hatched chick’s intestinal tract and spreading through the flock.

### The relative abundance of ASVs in the hen is not correlated with their transmission ability

The efficiently transmitted ASV unclassified *Lactobacillus* was found in high abundance in chick feces as well as in the hens’ fecal and jejunal samples, implying that it might be a symbiont vertically transmitting to progeny (Additional file 1: Figs. S3 and S4). In contrast, both the unclassified *Escherichia-Shigella* and the unclassified *Enterococcus* were found in low abundance in the hen fecal and internal samples, implying that an adapted opportunist label might better describe these two ASVs. Further analysis of the relative abundance in the hens of ASVs with different transmission abilities showed that most of the relative abundance in the hens represented ASVs that either did not transmit or poorly transmitted to chicks (Additional file 1: Fig. S3), based on 16S rRNA gene sequencing. Furthermore, a direct comparison between the transmission score and relative abundance in hen samples (feces, cecum, jejunum and magnum) showed no correlation between the two (data not shown). To conclude, transmission was not correlated with relative abundance in the hens.

### Non-shared ASVs poorly transmitted to chicks

The above results show that the transmission of hen bacteria to chicks is inefficient, suggesting that environmental opportunists might be able to fill the gap. Indeed, a very large number of ASVs found in chick fecal samples but not hen fecal samples using 16S rRNA gene sequencing using our primer set were observed (Fig. 3A). Furthermore, the number of chick ASVs not shared with the hens increased over time (Fig. 3C). A small number of these ASVs were observed in internal hen samples, implying that these were shared ASVs that were below the detection limit in hen fecal samples (data not shown). Of note, an analysis of the transmission score distribution showed that 98% of these non-shared chick ASVs were poorly transmitted to chicks, with a transmission score under 30% (Fig. 4B). To conclude, many non-shared ASVs were found in the chicks, but these were mostly poorly transmitted.

### Interventions reduced ASV transmission to chicks

**Fig. 6:**
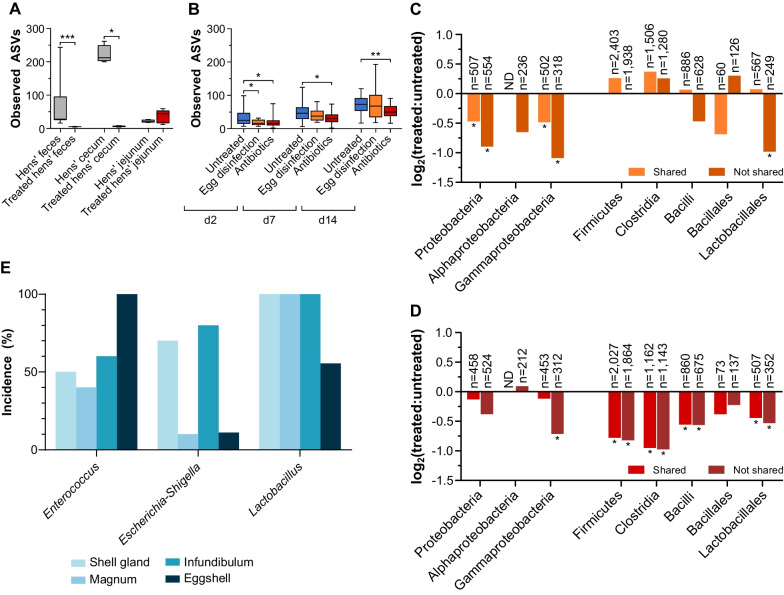
Analysis of interventions aimed at disrupting vertical transmission. **A** Number of observed ASVs in treated and untreated hen samples. Box plots are presented (centerline, median; box limits, upper and lower quartiles; whiskers, min and max values). Two-tailed Mann–Whitney test of each antibiotic-treated vs. untreated samples of the same type; hens’ feces n = 24, treated hens’ feces n = 4, hens’ cecum n = 4, treated hens’ cecum n = 4, hens’ jejunum n = 4, treated hens’ jejunum n = 5; *p < 0.05, ***p ≤ 0.001. **B** Number of observed ASVs in treated and untreated chick samples. Box plots are presented (centerline, median; box limits, upper and lower quartiles; whiskers, min and max values). Kruskal–Wallis test of each treated group vs. the untreated group; untreated d2 n = 87, egg disinfection d2 n = 22, antibiotics d2 n = 20; untreated d7 n = 89, egg disinfection d7 n = 20, antibiotics d7 n = 19; untreated d14 n = 86, egg disinfection d14 n = 22, antibiotics d14 n = 20; *p < 0.05, **p ≤ 0.01. **C**, **D** Treatment effect on the transmission of specific phylogenetic groups. **C**-disinfection. **D**-antibiotic cocktail. For both **C** and **D**, occurrences in chicks’ fecal samples of ASVs (either shared with hens’ feces or absent from hens’ samples) from each phylogenetic group were summed for treated and untreated chick samples and normalized to the number of samples in each treatment group (C-untreated n = 74, treated n = 64; D-untreated n = 69, treated n = 59). Log_2_ transformation of the fold change (treated:untreated) ratio is presented. Phylogenetic groups with at least 60 occurrences are presented. The number of occurrences in each phylogenetic group is indicated above the bar. To infer significance, 1,000 random permutations were generated by shuffling the treatment labels and recalculating the ratios. A ratio was considered significant if it was more than two standard deviations from the mean of the random permutations. **E** Presence of live bacterial cells of the top three transmitted ASVs in adult hens’ reproductive tract and on eggshells. For shell gland, magnum and infundibulum n = 10 each; for eggshell n = 9

To try and sever vertical transmission, two interventions were performed. In round 2, half of the eggs were sprayed with a disinfectant, Virocid, before incubation. A 38.5- and 40.4-fold reduction in CFU counts after disinfection was found in aerobic and anaerobic conditions, respectively (Additional file 1: Fig. S5). In round 3, half of the hens were treated with an antibiotic cocktail for two weeks, including during the week of egg collection. The antibiotic cocktail significantly reduced the richness of treated hens’ cecal and fecal samples (Fig. 6A) and modulated the composition of all hen samples (Fig. 2B, Additional file 1: Fig. S6 and Additional file 3: Table S4). The relative abundance in treated samples of two of the efficiently transmitted ASVs, the unclassified *Enterococcus* and *Escherichia-Shigella*, was increased (Additional file 1: Fig. S4), implying that they are adapted to the antibiotic-intensive commercial environment. The third efficiently transmitted ASV, the unclassified *Lactobacillus*, was not affected by antibiotic treatment. Thus, the antibiotic cocktail had a profound effect on the treated hens’ microbiota but did not reduce the abundance of the top three candidates for vertical transmission.

The fecal microbiota of egg-disinfection chicks showed a drop in richness only in day 2 samples, while the feces of chicks derived from antibiotic-treated hens showed a drop in richness at all three time points (Fig. 6B). A comparison by Jaccard index showed that the fecal microbiota of egg-disinfection chicks and chicks from antibiotic-treated hens was different from that of untreated chicks from the same round for all three time points (Additional file 1: Fig. S7). While the number of shared ASVs was only significantly reduced by the antibiotic treatment of the hens on day 14, the amount of non-shared ASVs was significantly reduced by both treatments at almost all time points (two-tailed one-sample t-test *p* < 0.05, untreated actual mean n = 3, Fig. 3B and C). An analysis of transmission scores showed a reduction in the number of non-shared ASVs with a score of < 10% in egg-disinfection chicks (Fig. 4B, two-tailed one-sample t-test *p* = 0.0079, untreated actual mean n = 3). The number of shared and non-shared ASVs in chicks derived from antibiotic-treated hens with transmission score < 10% was reduced (Fig. 4B, two-tailed one-sample t-test *p* = 0.0222, 0.0027 respectively, untreated actual mean n = 3), and more hen ASVs were never identified in chicks (transmission score = 0; Fig. 4A, two-tailed one-sample t-test *p* = 0.0045, untreated actual mean n = 3). To conclude, these effects caused by the interventions imply the existence of vertical transmission from hens to chicks via the egg.

To understand how specific phylogenetic groups were affected by egg disinfection and antibiotic treatment, the total number of occurrences of ASVs from each phylogenetic group in chick fecal samples was counted in treated and untreated chicks, and the ratio between them was calculated. This analysis showed that the antibiotic treatment had a broad effect, while egg disinfection did not affect Clostridia and Bacillales (Fig. 6C and D). As these two phylogenetic groups contain many spore formers, it can be hypothesized that this is the reason they were not affected. Finally, an analysis of the intermediate and efficiently transmitted ASVs showed that while a few ASVs seem to have been affected by the interventions, most were not affected (Fig. 5). To conclude, at least some percentage of the ASVs that transmit to chicks, including both shared and non-shared ASVs, utilize the egg to reach the chicks.

### Efficiently transmitted ASVs maintain their viability in hens’ reproductive tracts and on eggshells

The interventions described above did not disrupt the transmission of the three efficiently transmitted ASVs, which are the top candidates for vertically transmitted bacteria. To further investigate whether these three ASVs were using the egg for vertical transmission, the presence of viable bacteria on eggshells and in the reproductive tracts of a separate group of broiler breeders was determined. All three ASVs were cultured both from eggshell samples and from reproductive tract samples (Fig. 6E). The *Lactobacillus* was cultured from all reproductive tract samples as well as approximately half of the eggshell samples. The *Enterococcus* was cultured from 15 of 30 reproductive tract samples and from all nine eggshell samples. Finally, the *Escherichia-Shigella* was cultured from 70 and 80% of the shell gland and infundibulum samples, respectively, but from just one magnum and one eggshell samples. In conclusion, all three excellent colonizers were found to be viable in reproductive tract and eggshell samples, further supporting the hypothesis of vertical transmission through the egg. However, differences in their incidence between the sample types suggest that different routes might be used to reach the chicks.

## Discussion

This study demonstrates that under rearing conditions in which there is no interaction with adults, the representation of adult bacterial strains in chicks is very low, implying that vertical transmission is limited. These conditions closely represent the growth practices of many commercial operations commonly practiced for reasons of biosecurity. Moreover, in many commercial operations, eggs are treated with disinfectants which reduce vertical transmission even further. This data is further corroborated by studies showing that early exposure of chicks to the gut contents of adults or to live adults enables the transmission of many bacterial strains [4, 13, 14]. Indeed, the fact that artificial exposure enables the colonization of many bacterial strains implies that the limited transmission found in our data is not a result of the gut environment of newly hatched chicks not being able to support these bacteria (due to lack of gut maturation or bacterial succession) but likely because of a lack of exposure. Thus, it seems that the effort to inhibit the transfer of pathogens also disrupts the vertical transfer of commensal gut microbes.

As the gut microbiota plays a key role during early life development [1, 2], and as this report shows, vertical transmission is limited due to commercial conditions, it can be hypothesized that many microbiota functions are impaired. One such function of the gut microbiota for the host is protection against gut pathogens. Young chicks are sensitive to gut pathogens, such as the zoonotic pathogen *Salmonella*, with financial implications in commercial production [28]. It has been shown that exposure to live adults or to the gut content of adults renders chicks resistant to *Salmonella* infection [13, 14]. It has also been shown that as they age, chicks become resistant to *Salmonella*, likely because they acquire commensal gut bacteria which protect them [13]. The results presented in this study, showing minimal vertical transmission in commercial growth conditions, complete this picture and imply that modern growth practices may be the cause of sensitivity to, at least, *Salmonella* infection.

While this work showed that most hen bacterial strains did not efficiently transmit to chicks, it also revealed that some vertical transmission is still likely to occur in these conditions, where there is no interaction with adults. Based on 16S rRNA gene sequencing, we found that three ASVs present in hens did reach the chicks efficiently, approximately 20 more had intermediate transmission ability and many more poorly transmitted or did not transmit to chicks. All shared intermediate and efficiently transmitted ASVs were consistently so in all three rounds. Thus, a specific set of bacterial strains were consistently transmitted to chicks, at least when the same hens are involved.

The results of this work raise an interesting question: how do hens receive their gut microbiota? It can be hypothesized that while vertical transmission through the egg or the external environment is inefficient, it is sufficient, given the longer life spans of hens. In the present experiment, many of the shared bacterial strains did make it to a few of the progeny. It can be hypothesized that as the flock ages, bacterial strains would spread through the flock, giving each bird a full and complete gut microbiota population as it matures. To conclude, in commercial operations in which there is no contact with adult hens, the most efficient mechanism of vertical transmission is lacking. In these conditions, inefficient vertical transmission through the egg or the environment becomes the main mechanism of vertical transmission.

We performed two interventions aimed at disrupting any vertical transmission that may take place via the egg. Both interventions resulted in a decrease in the transmission of ASVs shared with hens. A surprising finding in this study is that interventions designed to disrupt vertical transmission had a large effect on bacterial strains that were found in the chicks but not in the hens. Such strains are viewed as environmental opportunists – bacterial strains that are not specifically adapted to the gut of chickens but colonize this niche because it is relatively empty. Environmental opportunists are thought to come from feed, cage surfaces or even dust particles. In this study, each of the two interventions significantly reduced the transmission of non-shared ASVs. This implies that a considerable proportion of environmental opportunists utilize the egg for transmission to chicks. This portion is likely larger than what was observed in this study, as both interventions performed were not optimal – Virocid spraying reduced the numbers of bacteria on the eggshell but did not sterilize it, and the application of the antibiotic cocktail had little effect on many intermediate and efficiently transmitted ASVs, including non-shared strains, implying that many have inherent antibiotic resistance. One possibility is that these non-shared strains do come from the hen’s body, perhaps from the skin or cloaca or are even found at very low levels, below the detection level, in the gut and feces and that a more appropriate label would be adapted opportunists. To conclude, it is likely that a large portion of the non-shared bacterial strains colonizing the chicks are not environmental opportunists per se but are rather chicken-adapted opportunists that are not able to forge a niche for themselves in the adult chicken gut. These adapted opportunists take advantage of the newly hatched chick’s relatively empty gut and then are able to survive on or in the hen at low numbers and to be vertically transmitted through the egg to the next generation.

Moreover, we showed that the three efficiently transmitted ASVs are present and alive both in samples of hens’ reproductive tracts and in eggshell samples by cultivating them directly from these samples. This indicates that vertical transmission via the egg is indeed a mechanism for vertical transmission, even if not efficient. These results are in agreement with previous studies that showed that gut material is sampled into the female reproductive tract, implying that gut bacteria were in the right place to integrate into forming eggs [10], as well as studies identifying bacterial DNA in embryos [9, 11, 29] and showing some resemblance between the microbiota of hens and chicks [9, 11]. This indicates that at least these three ASVs utilize the egg for vertical transmission. Interestingly, their distribution in the samples was different. The *Lactobacillus* was found in all 30 samples of the reproductive tract, including the infundibulum, magnum and shell gland. This suggests that the *Lactobacillus* was adapted to the conditions in the reproductive tract and likely in the egg white, which is secreted in the magnum section of the reproductive tract. The *Lactobacillus* was found only in half of the eggshell samples, indicating that it was less adapted to this environment. Conversely, the *Enterococcus* was found in all eggshell samples but only in half of the reproductive tract samples, implying that it was more adapted to the eggshell environment. Thus, it is possible that while all three efficiently transmitted ASVs use the egg as a vehicle for vertical transmission, they utilize different parts of the egg. To conclude, our results show that some bacterial strains utilize the egg for vertical transmission, and thus the egg is not only a source for nutrients for the developing chick but also the source for some gut commensals.

Finally, it is important to acknowledge the limitations of this work. More than one hundred chicks were studied, and each chick was sampled three times. However, only ten hens were included in this study, although these hens seem to be representative according to microbiota composition [10, 11, 30, 31]. Furthermore, the results presented here show that most of the shared ASVs were only identified in a few chicks. The most likely hypothesis explaining these results is that chicks were simply not exposed to these ASVs. This hypothesis is supported by published work showing that artificial exposure to many adult-derived bacteria resulted in colonization [4, 13, 32]. However, it is also possible that non-colonized chicks were exposed to these ASVs but were somehow resistant to colonization. Another option is that these ASVs were present in the chicks but were below the detection limit in the feces. This is true for all sample types and is a known limitation of 16S rRNA gene sequencing. Indeed, we noted a few ASVs that were found in hen internal samples but were not found in hen fecal samples. Furthermore, the fact that the interventions had an effect on non-shared ASVs implied that these ASVs might have come from the hens but were below the detection limit in hen samples. Finally, as different gut sections empty periodically and harbor different microbial populations, a prominent member of one section may be poorly represented in the feces and not be detected using our methodology. For this reason, many of our analyses were based on the whole chick and hen population.

## Conclusions

We show here that some bacterial strains utilize the egg for vertical transmission and that the egg is a source of some commensal gut bacteria for the chick. Conversely, we also show that vertical transmission in commercial conditions in which there is no contact between chicks and adults is very inefficient. On the whole flock level, while coupled with horizontal transmission through the flock over time, it is likely sufficient for the transfer of the current modern chicken microbiota. However, at the single-chick level, this results in a long delay in the acquisition of an adult gut microbiota. This might explain why chicks are sensitive to gut pathogens and why they become resistant after exposure to an adult’s cecum content. These results imply that other gut functions might likewise be suboptimal and that artificial exposure to adult bacteria might correct these problems.

### Supplementary Information

**Additional file 1**. **Figure S1:** Correlation between transmission score and relative abundance in chick fecal samples; **Figure S2:** Heatmap of the dominant ASV in each sample; **Figure S3:** Cumulative relative abundance of ASVs in hens, binned by their transmission scores in chicks; **Figure S4:** Relative abundance of the three efficiently transmitted ASVs in treated and untreated hens and chicks; **Figure S5:** Effect of disinfection on the bacterial load of eggshell surfaces; **Figure S6:** Antibiotic treatment disrupts hen microbiota; and **Figure S7:** PCoAs using Jaccard index of treated vs. untreated chicks of the same round in each time point.

**Additional file 2**. Raw data of all samples and all ASVs and the number of reads.

**Additional file 3**. **Table S1:** ANOSIM between different age groups; **Table S2:** ANOSIM of chicks between different rounds; **Table S3:** ANOSIM of untreated hen samples in different rounds; and **Table S4:** ANOSIM between treated and untreated hens.

## Data Availability

The datasets generated and analyzed during the current study are available in the SRA repository under the accession number PRJNA885292.
