# Supplementary material for: Vertical transmission of gut bacteria in commercial chickens is limited
Source: Anim Microbiome. 2023 Oct 10;5:50. doi: 10.1186/s42523-023-00272-6 (PMC10566040; doi:10.1186/s42523-023-00272-6)
Supplement: Supplementary file 1 — Additional file 1. Figure S1: Correlation between transmission score and relative abundance in chick fecal samples; Figure S2: Heatmap of the dominant ASV in each sample; Figure S3: Cumulative relative abundance of ASVs in hens, binned by their transmission scores in chicks; Figure S4: Relative abundance of the three efficiently transmitted ASVs in treated and untreated hens and chicks; Figure S5: Effect of disinfection on the bacterial load of eggshell surfaces; Figure S6: Antibiotic treatment disrupts hen microbiota; and Figure S7: PCoAs using Jaccard index of treated vs. untreated chicks of the same round in each time point. [file 42523_2023_272_MOESM1_ESM.pdf]

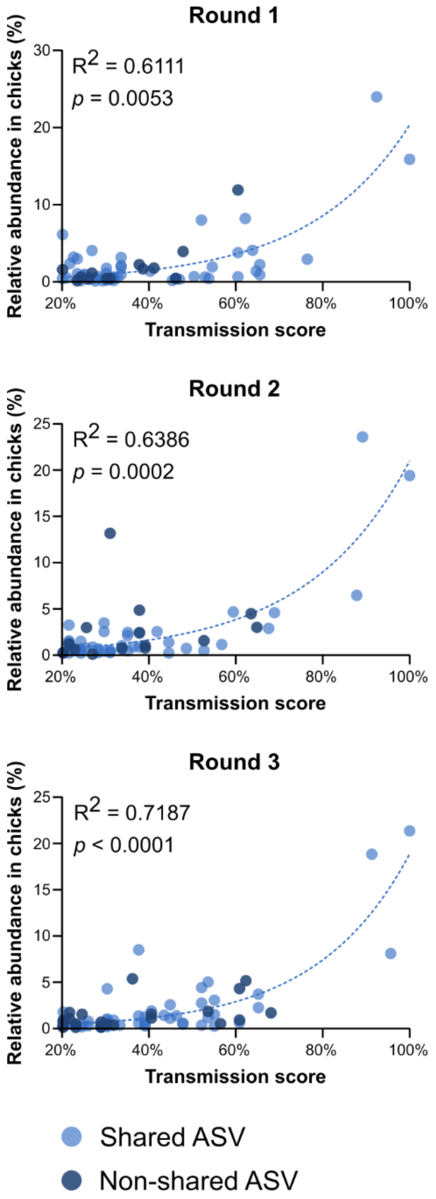

<u>Figure S1</u>: Correlation between transmission score and relative abundance in chick fecal samples. The transmission score of each ASV was plotted against its average relative abundance in untreated chicks. Light blue dots represent ASVs that appear in hen feces samples, and dark blue dots represent ASVs that appear only in chicks. Only ASVs with a transmission score of ≥20% were included in the analysis. Non-linear fit R^2 and Spearman correlation *p-value* are presented for each panel.

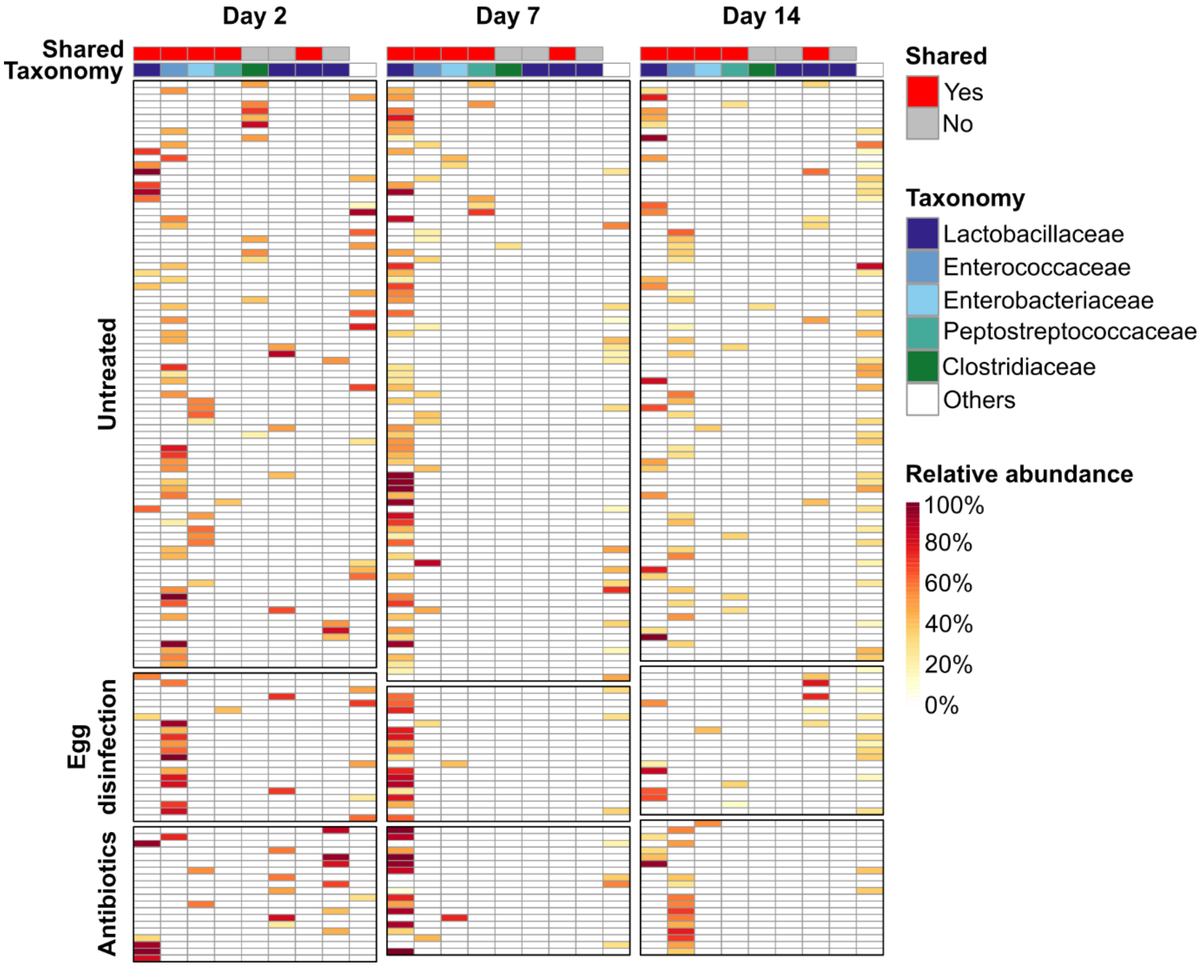

Figure S2: Heatmap of the dominant ASV in each sample. Dominance was defined as encompassing more than 30% of the reads of the specific sample. Only ASVs that were the dominant ASV in ≥10 samples are presented (a total of 8 ASVs). Each column represents a single ASV, and the column denoted as "others" encompasses all ASVs that were dominant in <10 samples.

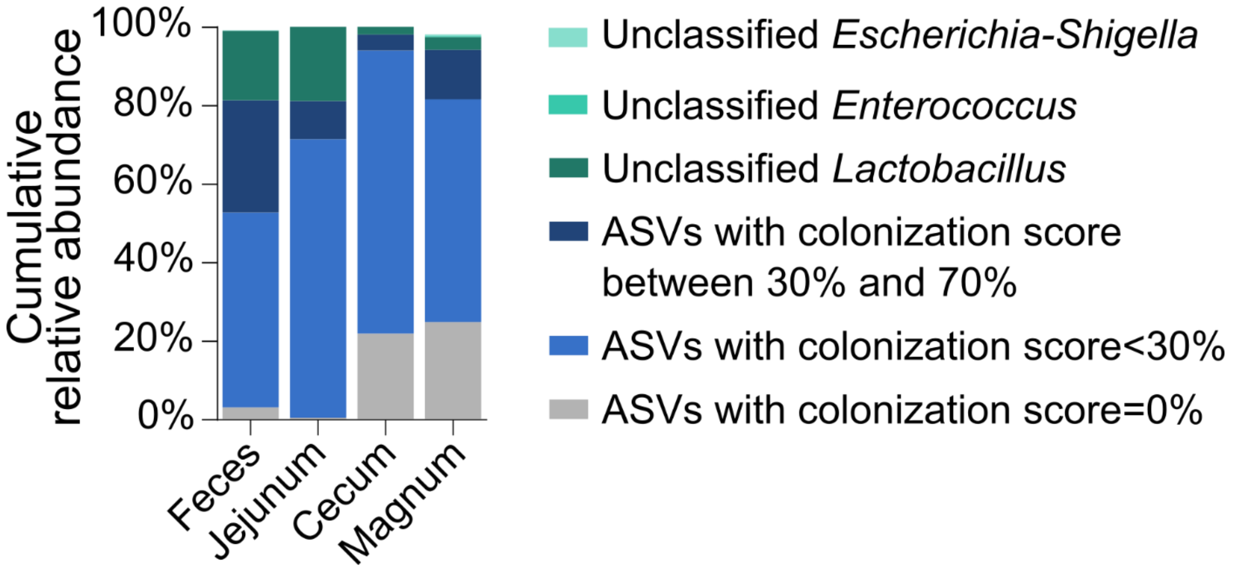

<u>Figure S3</u>: Cumulative relative abundance of ASVs in hens, binned by their transmission scores in chicks. The top three transmitting ASVs (unclassified *Escherichia-Shigella*, unclassified *Enterococccus* and unclassified *Lactobacillus*) are presented separately.

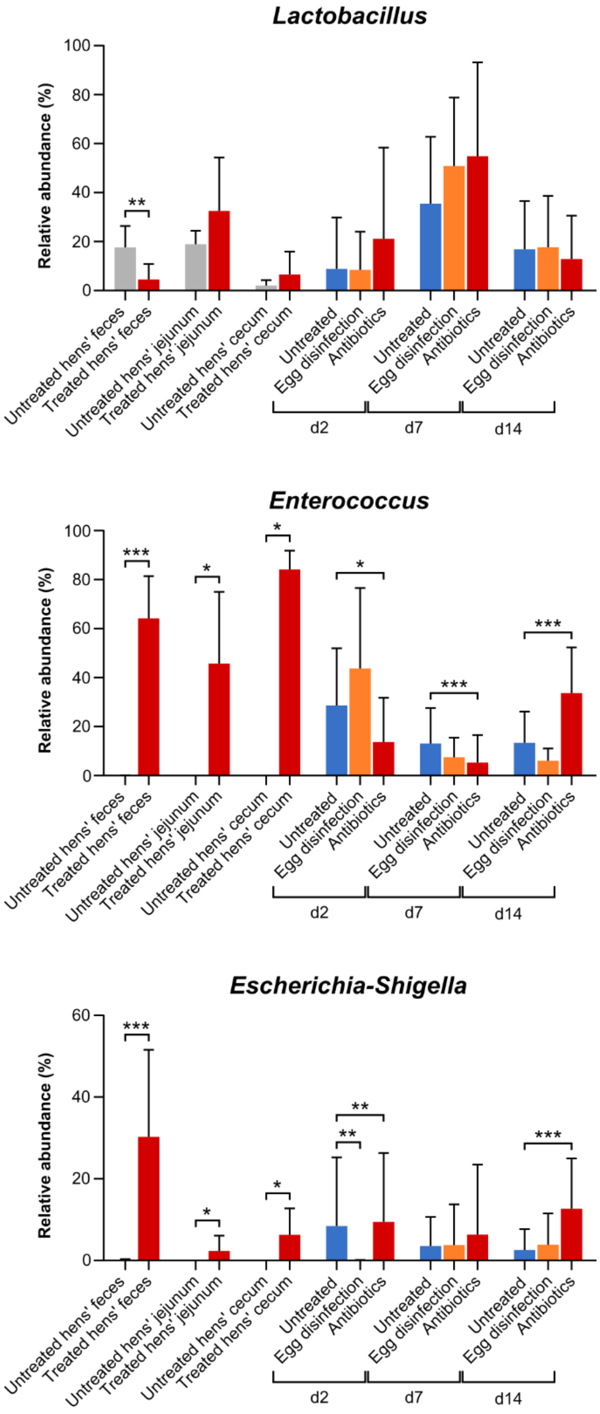

Figure S4: Relative abundance of the three efficiently transmitted ASVs in treated and untreated hens and chicks. The difference between treated and untreated hen samples was tested by Mann-Whitney test, and the difference between treated and untreated chick samples for each day was tested by Kruskal-Wallis test; * $p < 0.05$, ** $p \leq 0.01$, *** $p \leq 0.001$.

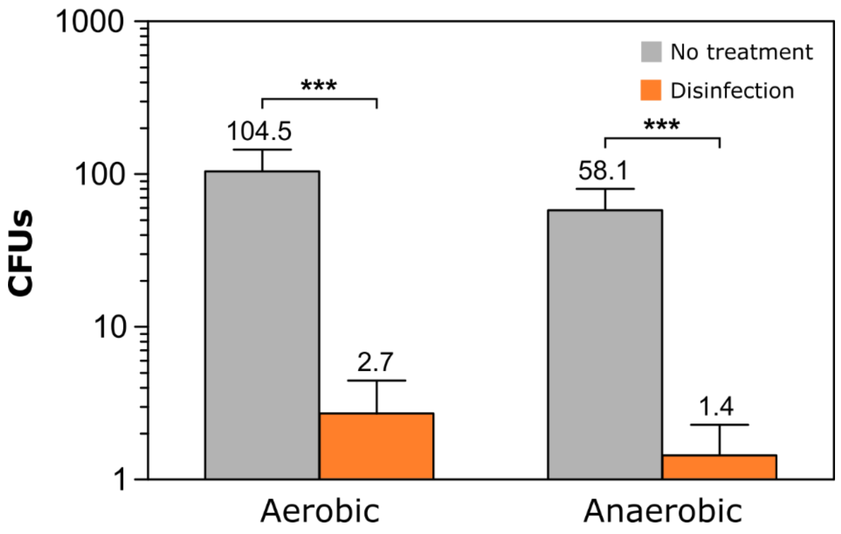

<u>Figure S5</u>: Effect of disinfection on the bacterial load of eggshell surfaces. A 4 cm^2 area of untreated and disinfected eggshells was swabbed and resuspended in 600 µl of D/E buffer. 200 µl were plated on YCFA plates in aerobic and anaerobic conditions. Mann-Whitney test; *** $p < 0.001$.

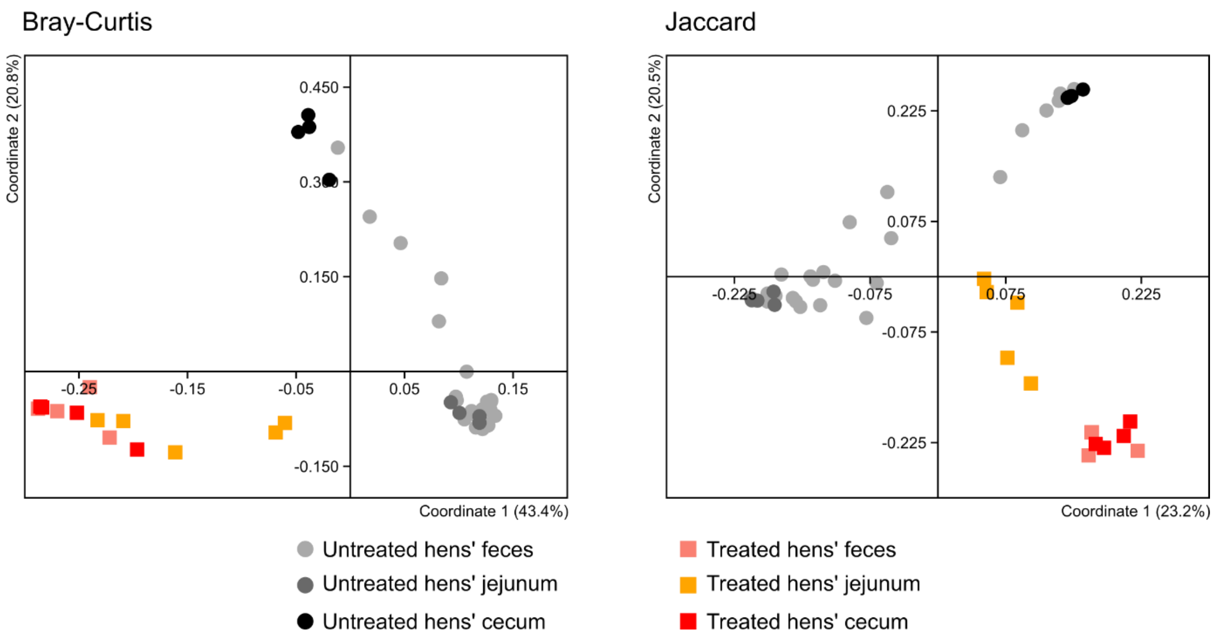

Figure S6: Antibiotic treatment disrupts hen microbiota. PCoA of untreated and antibiotic-treated hens using Bray-Curits and Jaccard metrics.

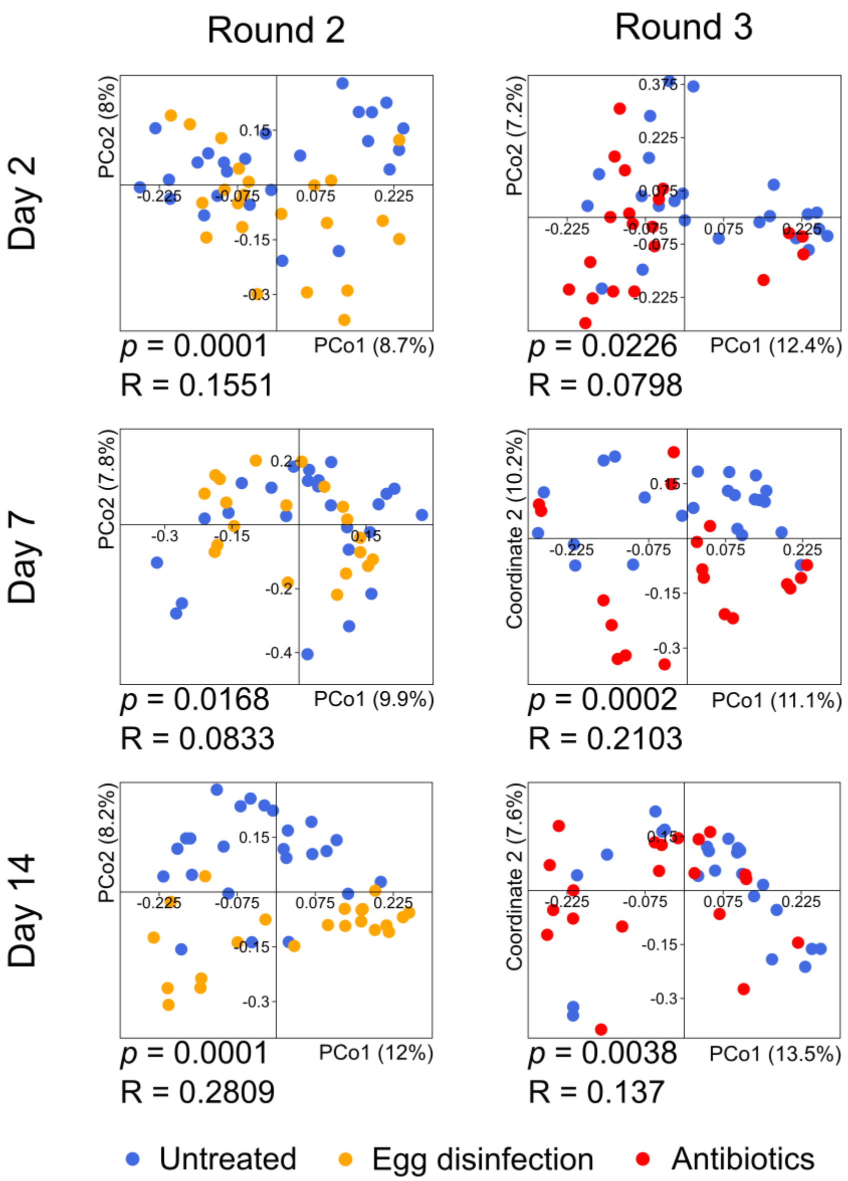

<u>Figure S7</u>: PCoAs using Jaccard index of treated vs. untreated chicks of the same round in each time point. ANOSIM *p* and R values are indicated for each panel.
