# Supplementary material for: Vertical transmission of gut bacteria in commercial chickens is limited
Source: Anim Microbiome. 2023 Oct 10;5:50. doi: 10.1186/s42523-023-00272-6 (PMC10566040; doi:10.1186/s42523-023-00272-6)
Supplement: Supplementary file 3 — Additional file 3. Table S1: ANOSIM between different age groups; Table S2: ANOSIM of chicks between different rounds; Table S3: ANOSIM of untreated hen samples in different rounds; and Table S4: ANOSIM between treated and untreated hens. [file 42523_2023_272_MOESM3_ESM.pdf]

Supplementary table S1: ANOSIM between different age groups.

		Round 1		Round 2		Round 3	
Group 1	Group 2	*p-value*	R value	*p-value*	R value	*p-value*	R value
Untreated hens	Day 2	0.0006	0.9373	0.0006	0.8887	0.0006	0.8635
	Day 7	0.0006	0.9098	0.0006	0.9037	0.0006	0.9018
	Day 14	0.0006	0.8699	0.0006	0.8559	0.0006	0.908
Day 2	Day 7	0.0006	0.2617	0.0006	0.5789	0.0006	0.3619
	Day 14	0.0006	0.7194	0.0006	0.7848	0.0006	0.6116
Day 7	Day 14	0.0006	0.3734	0.0006	0.3433	0.0006	0.2581

* Bonferroni-corrected *p-values* are presented

Supplementary table S2: ANOSIM of chicks between different rounds.

		Day 2		Day 7		Day 14	
Group 1	Group 2	*p-value*	R value	*p-value*	R value	*p-value*	R value
Round 1	Round 2	0.0003	0.6006	0.0003	0.3552	0.0003	0.3964
	Round 3	0.0003	0.5236	0.0003	0.43	0.0003	0.3812
Round 2	Round 3	0.0003	0.292	0.0138	0.104	0.0003	0.2751

* Bonferroni-corrected *p-values* are presented

Supplementary table S3: ANOSIM of untreated hen samples in different rounds.

		Bray-Curtis		Jaccard	
Group 1	Group 2	*p-value*	R value	*p-value*	R value
Round 1	Round 2	0.0894	0.1053	0.2037	0.1076
	Round 3	1	-0.102	1	0.00147
Round 2	Round 3	0.4485	0.1725	0.4344	0.176

* Bonferroni-corrected *p-values* are presented

Supplementary table S4: ANOSIM between treated and untreated hens.

	Bray-Curtis		Jaccard	
Site	*p-value*	R value	*p-value*	R value
Feces	0.0002	0.9775	0.0002	0.7609
Jejunum	0.0079	0.95	0.0079	0.8781
Cecum	0.029	1	0.03	1
